# Integrating Transdiagnostic and Biopsychosocial Approaches to Move Beyond Categorical Diagnoses in Neurodevelopmental Disorders: A Perspective Review

**DOI:** 10.1002/pchj.70107

**Published:** 2026-05-28

**Authors:** Duo Liu, Shu Liu, Lirong Luo

**Affiliations:** ^1^ Department of Special Education and Counselling The Education University of Hong Kong Hong Kong SAR China

**Keywords:** biopsychosocial model, children with special educational needs, neurodevelopmental disorders, transdiagnostic approach

## Abstract

Neurodevelopmental disorders (NDD) represent a heterogeneous group of conditions that are thought to be related to impaired brain development, which often leads to significant functional difficulties across various domains. According to the Diagnostic and Statistical Manual of Mental Disorders (DSM) and the International Statistical Classification of Diseases and Related Health Problems (ICD), conventional diagnostic frameworks mainly focus on categorical classifications for identifying and supporting children with NDD. Despite their merit throughout the history of special and inclusive education, various stakeholders revealed the imperfections of this categorical diagnostic system. One aim of this perspective review, thus, is to promote a transdiagnostic approach for understanding NDDs as a possible supplementation of the current categorical diagnosis system. At the same time, there is limited relevant research on psychological and ecological domains beyond the symptomatic and neurocognitive domains of NDD. We propose to extend the transdiagnostic approach within the framework of a biopsychosocial approach that integrates neurocognitive, psychological, and socioecological factors and aims to enhance the understanding of NDD and the broader context of inclusive education.

## 1 Introduction

Neurodevelopmental disorders (NDD) were defined as conditions involving impairment of the neurological system and the brain, ranging from specific to global impairment in functioning (American Psychiatric Association 2013; Thapar et al. 2017). According to the *Diagnostic and Statistical Manual of Mental Disorders*, 5th Edition (*DSM‐5*), NDD are classified into Intellectual Disability (ID), Communication Disorders (CD), Autism Spectrum Disorder (ASD), Attention‐Deficit/Hyperactivity Disorder (ADHD), Specific Learning Disorder (SpLD), Motor Disorders, and Other NDDs (American Psychiatric Association 2013). While these diagnostic categories have served as important clinical and research tools, growing evidence highlights their significant limitations in capturing the complex reality of neurodevelopmental diversities (Astle et al. 2022; Dalgleish et al. 2020). Therefore, this article aims to promote integrating the transdiagnostic approach and biopsychosocial model as a promising direction to supplement the current diagnostic practice and inform the development of inclusive education.

The field of NDD has traditionally adhered to the widespread categorical diagnostic approach, such as DSM‐5 (American Psychiatric Association 2013) and the International Statistical Classification of Diseases 11th Revision (ICD‐11, World Health Organization 2019), which understands NDD through classifying conditions from different dimensions based on specific symptomatology. This categorical approach has a long history and is a well‐established system recognized by various disciplines and countries. More importantly, it plays a crucial role in shaping the way people identify and address health conditions (Dalgleish et al. 2020). However, such a diagnostic system faces at least three critical issues. First, the reliance on categorical thresholds results in substantial numbers of children with symptoms failing to meet diagnostic criteria (Astle et al. 2022). This is particularly evident when identifying children with milder forms of NDD, as many who exhibit symptoms fail to receive an official diagnosis due to stringent diagnostic criteria or systemic barriers related to racial, ethnic, and socioeconomic factors (Huang et al. 2013).

For example, a study by Russell et al. (2014) found that approximately 50% of children with ASD traits in the United Kingdom (UK) did not meet the threshold for a formal diagnosis, leaving them without access to necessary support. Similarly, in the context of ADHD, studies have highlighted that children from marginalized or low‐income backgrounds are less likely to receive a diagnosis despite displaying clear symptoms (Danielson et al. 2018; Sayal et al. 2018). In China, the prevalence of ASD is notably lower than global prevenlence (Sun et al. 2019; Wang et al. 2019; Zhang and Spencer 2015), partly due to the conservative diagnostic practices of Chinese psychiatrists, who often opt for labels such as “autistic tendency” rather than an official diagnosis (Huang et al. 2013).

As a result, children with milder NDD characteristics frequently miss out on formal diagnoses and, consequently, the official support they need. This is particularly problematic under the current major inclusive education policy framework, which only allows children with official diagnoses to access systemic support and resources (Florian and Camedda 2020; Norwich 2014). This neglect has significant consequences. Research indicates that undiagnosed children with NDD symptoms are at high risk of academic underachievement, social exclusion, and mental health challenges (Emerson and Hatton 2007; Totsika et al. 2011). For instance, a longitudinal study by Totsika et al. (2011) found that children with undiagnosed developmental difficulties were more likely to experience bullying and emotional distress compared to their peers with formal diagnoses. Parents/caregivers of such children may suffer extra stress and difficulties because their child's problems were not fairly treated (Cullen and Lindsay 2019).

Second, children with the same diagnostic label often exhibit significant variability in the scope, nature, and impact of their symptoms (Kofler et al. 2019; Masi et al. 2017). Heterogeneous profiles are prevalent across different NDDs, meaning that the diagnostic label alone may not fully capture the diverse characteristics of individuals within the same category. For example, children with ASD display a wide range of language‐related profiles, from nonverbal communication to advanced reading skills (Brown et al. 2013), as well as variability in cognitive functioning (Zheng et al. 2020), sensory processing (Tillmann et al. 2020), and co‐occurring psychological conditions (Simonoff et al. 2008). Similarly, children with ADHD exhibit diverse clinical profiles and etiological risk factors (Luo et al. 2019). Beyond core symptoms such as inattention, executive dysfunction, and working memory deficits, children with ADHD may also experience challenges in cognitive flexibility, emotional regulation, and social functioning. Neurobiological studies further highlight this heterogeneity, revealing varied electroencephalography (EEG) patterns and magnetic resonance imaging (MRI) findings among individuals with ADHD (Nigg, Karalunas, et al. 2020; Nigg, Sibley, et al. 2020).

SpLDs, such as dyslexia and dyscalculia, also demonstrate significant heterogeneity in their manifestations (Grigorenko et al. 2019). For instance, dyslexia can present as difficulties in phonological processing, semantic/morphological processing, or visual‐orthographic processing (O'Brien et al. 2012; Shu et al. 2005), while dyscalculia may involve challenges in number sense, arithmetic, or mathematical reasoning (Butterworth et al. 2011; Träff et al. 2017). This variability makes it difficult to establish uniform criteria for identifying SpLDs, as the same diagnosis can encompass vastly different learning profiles (Peters and Ansari 2019). Consequently, relying solely on group‐level performance to understand SpLD can obscure critical individual differences, limiting the development of targeted interventions and support strategies.

The third issue researchers, clinicians, and educators face when using these categorical diagnostic criteria is the high rate of comorbidity among NDD (Astle et al. 2022). Comorbidity of two or more NDD is common. For example, a study examining 74 children and adolescents with NDD found that 47% of participants had ID alongside ASD, ADHD, or both, while 11% had ASD comorbid with ADHD (Márquez‐Caraveo et al. 2021). ASD is frequently found to co‐occur with other neurodevelopmental and psychological conditions, including ADHD, anxiety, depression, and sleep disorders (Bougeard et al. 2021). Overlapping cognitive deficits have also been reported in ASD and ADHD (Doi et al. 2022), and evidence for shared brain network dysfunction in these conditions has been identified (Kernbach et al. 2018). The overlapping profiles and high comorbidity rates among NDD make it challenging for researchers to isolate the impact of a specific disorder on learning outcomes. Similarly, a high co‐occurrence rate of dyslexia and dyscalculia among children with SpLD has been reported, leading to debates over whether SpLD stems from a core deficit or a general deficit (Peters and Ansari 2019). Furthermore, SpLD is often comorbid with ASD and ADHD (Kessler et al. 2006), further complicating the diagnostic picture. Relying solely on categorical diagnostic criteria may fail to capture the complexity of comorbid conditions, leading to a loss of critical information about individual variation, which is essential for effective educational and clinical interventions.

Therefore, a transdiagnostic approach is needed to address the limitations of the current diagnostic system. This approach should consider the three key issues outlined above: (1) the inadequacy of diagnostic criteria in identifying children with milder NDD symptoms, (2) the significant heterogeneity within diagnostic categories, and (3) the high rates of comorbidity among NDD. Moving beyond categorical diagnoses and focusing on shared domains or factors, a transdiagnostic framework can deepen our understanding of how these factors interact to influence the learning and development of children with NDD (Astle et al. 2022). Such an approach would not only provide a more nuanced understanding of individual differences but also inform the development of targeted, evidence‐based interventions that address each child's unique needs.

## 2 Current Transdiagnostic Study

The transdiagnostic approach has been widely used in the mental health field, including identifying factors or biomarkers associated with symptoms and development, evaluating transdiagnostic treatments, and providing insight into clinical diagnosis and subtyping of mental health issues (Dalgleish et al. 2020; Schaeuffele et al. 2024). The types of research questions and findings can shed light on NDD research. This section summarized the application of the transdiagnostic approach in mental health, followed by a review of the literature on transdiagnostic studies of NDD.

### 2.1 Transdiagnostic Approach in the Field of Mental Health

Early work underlying the transdiagnostic approach in the field of mental health highlighted shared processes, such as attentional biases, rumination, and emotional dysregulation, across anxiety, depression, and other emotional disorders (Harvey et al. 2004; Ehring and Watkins 2008). These findings underscore the role of transdiagnostic processes in explaining comorbidity and heterogeneity in mental problems. Neuroimaging studies further advanced this approach by identifying shared neural circuits and biomarkers across psychiatric disorders. For example, McTeague et al. (2017) found common patterns of neural dysfunction in reward processing and emotional regulation across mood, anxiety, and psychotic disorders. Similarly, Sasabayashi et al. (2021) identified atypical neural circuits shared across schizophrenia, bipolar disorder, major depressive disorder, and ASD. Large‐scale studies have reinforced the utility of transdiagnostic dimensions in understanding psychopathology. Quattrone et al. (2019) identified factors such as cognitive deficits and emotional dysregulation that contribute to symptoms across affective and non‐affective psychosis. Caspi et al. (2014) proposed the “p‐factor,” a general psychopathology factor capturing shared variance across mental health disorders. The transdiagnostic approach has also informed the development of treatments targeting shared mechanisms. Transdiagnostic cognitive‐behavioral therapy (CBT) has proven effective for anxiety and depression by addressing common processes like avoidance and maladaptive thinking (Barlow et al. 2017; Norton and Paulus 2017). Similarly, Sherman and Ehrenreich‐May (2020) demonstrated the efficacy of transdiagnostic treatments for emotional disorders in adolescents.

In summary, the transdiagnostic approach has advanced mental health research by shifting focus from categorical diagnoses to shared mechanisms and dimensions. These insights set the stage for applying this approach to NDD, where comorbidity and heterogeneity are equally prevalent.

### 2.2 Transdiagnostic Approach in the Studies of NDD

Recently, the transdiagnostic approach has been adopted in a number of NDD studies. A leading example of this paradigm is the work from the Centre for Attention Learning and Memory (CALM). The CALM project (Holmes et al. 2019) was specifically designed to investigate children with developmental difficulties using a transdiagnostic framework. To differentiate dimensions related to learning challenges, the project focuses on the cognitive factors closely linked to learning in children with varied NDD diagnostic statuses. Participants include children struggling with attention, learning, or memory, alongside typically developing peers. The study collected a rich, multi‐level dataset, including children's cognitive abilities and learning outcomes in language and math, parent questionnaires on family background, behavior, and communication, as well as MRI brain imaging and DNA samples.

Subsequent data‐driven analyses of this cohort have robustly validated the transdiagnostic approach. For instance, in a factor analysis of 805 children, Holmes et al. (2021) identified three core transdiagnostic dimensions, that is, difficulties in phonological processing, processing speed, and executive functioning, that captured shared variance across children with varied NDD presentations. Extending this work, Mareva et al. (2023) further delineated three subgroups based on language processing, pragmatic communication, and executive function profiles, demonstrating that these behavioral patterns, alongside white matter organization, better explained commonalities among children with heterogeneous symptoms than traditional diagnostic labels. Complementing these cognitive and behavioral findings, neuroimaging research within the CALM project has linked such profiles to brain organization. Jones et al. (2021), using resting‐state fMRI data from the same cohort, identified three distinct behavioral profiles characterized by principal difficulties with hot executive function (related to emotion and reward), cool executive function (related to cognitive control), and learning. While global brain organization did not differ between these groups, multivariate patterns of functional connectivity, particularly within networks supporting cognitive control, emotion, and social cognition, significantly distinguished the groups, revealing both general and specific neurodevelopmental risk factors in the functional connections.

Research from other groups provided further evidence supporting the application of the transdiagnostic approach in NDD studies. For example, Apperly et al. (2024) examined the latent factor structure of broader phenotype traits associated with six neurodevelopmental conditions in a representative sample of 995 UK adults. Their analyses revealed a general “*N* factor” accounting for the largest proportion of variance across all measures, alongside four specific factors corresponding to traits associated with autism, ADHD, cortical hyperexcitability, and dyslexia/developmental coordination disorder. This *N* + 4 structure suggests that broader phenotype scales measure both a general neurodiversity dimension and more condition‐specific variability, offering a powerful framework for understanding the complex patterns of co‐occurrence and heterogeneity inherent to neurodevelopmental conditions.

Azim et al. (2025) applied cluster and factor analyses to two large clinical datasets, namely the Australian Autism Biobank and the Child Behavior Research Clinic, to identify transdiagnostic domains in children. Their integrated findings revealed consistent patterns across communication, emotional regulation, social interaction, behavioral control, and cognitive functioning, supporting the development of a unified transdiagnostic assessment scale that captures overlapping characteristics across ASD, ADHD, and other neurodevelopmental conditions, rather than relying on individual diagnostic instruments.

Al‐Saoud et al. (2025) employed unsupervised machine learning (ML) on a large sample of over 1500 children and adolescents, identifying six distinct cognitive clusters based on performance in short‐term memory, reasoning, and verbal ability. Crucially, diagnostic status, that is, whether ADHD, ASD, or comorbid ADHD/ASD, showed no correspondence with cluster membership, highlighting the profound cognitive heterogeneity masked by categorical labels. In a clinically well‐characterized Mexican sample, Márquez‐Caraveo et al. (2021) applied latent class analysis to the Wechsler Intelligence Scale for Children, Fourth Edition (WISC‐IV) profiles and uncovered a four‐cluster solution to regroup the participants with ID, ASD, and/or ADHD. Children with the same diagnostic label often fell into different cognitive subgroups; for instance, those with ASD were distributed across three distinct classes based on their working memory and perceptual reasoning scores, underscoring the variability within a single diagnostic category.

Jacobs et al. (2021) identified four robust, transdiagnostic biotypes across children with ASD, ADHD, and obsessive‐compulsive disorder (OCD), characterized by distinct patterns of cortical thickness, subcortical volume, and symptomatology that were not discernible through categorical comparisons. Adding further weight to this literature, Cleary et al. (2026) applied heterogeneous mixture models to behavioral data from over 1700 participants, including typically developing children and those with ASD or ADHD. Their analysis revealed six distinct behavioral clusters with profiles ranging from low impairment to severe, cross‐domain difficulties. Notably, all six clusters were transdiagnostic, containing individuals from multiple diagnostic categories, and the most severely impaired cluster comprised exclusively ASD and ADHD participants, underscoring that symptom profiles often fail to align with expected diagnostic phenotypes.

To provide readers with a systematic overview of the key dimensions identified across transdiagnostic studies cited in this review, we summarize the main findings in Table 1. These findings underscore a critical paradigm shift: moving from a diagnosis‐oriented model to a transdiagnostic, mechanism‐focused approach is not merely a theoretical exercise but an empirical necessity. As noted by Finlay‐Jones et al. (2019), such a shift is pivotal for moving beyond descriptive labels to enable earlier identification of risk and, ultimately, the development of targeted interventions that address the specific neurocognitive and biological mechanisms driving an individual's challenges, rather than the heterogeneous constellation of symptoms that define their diagnosis. That is to say, the transdiagnostic framework is not merely a new system for “re‐categorizing” children with NDD. One of its defining features, that is, distinguishing it from conventional diagnostic approaches, is that its clustering is dimensional, probabilistic, and empirically derived (i.e., data‐driven), rather than predetermined by categorical labels. In this framework, the fit between individual cases and identified clusters can be optimized to better capture the heterogeneity within and across diagnostic groups. As a result, individual needs are more effectively embraced, a goal that traditional diagnostic approaches often struggle to achieve.

**TABLE 1:** Summary of key transdiagnostic dimensions identified in NDD studies.

Study	Sample	Methods	Key dimensions/subgroups identified	Main findings
Al‐Saoud et al. (2025)	1529 children and adolescents	Unsupervised machine learning (self‐organizing maps + k‐means clustering)	Six distinct cognitive clusters based on performance in: Short‐term memory Reasoning Verbal ability	Diagnostic status (ADHD, ASD, comorbid) showed no correspondence with cluster membership, highlighting profound cognitive heterogeneity masked by categorical labels
Apperly et al. (2024)	995 adults	Bifactor modeling	General “*N* factor” (accounting for largest proportion of variance across all measures) Four specific factors: ASD, ADHD, cortical hyperexcitability, dyslexia/DCD	*N* + 4 structure reveals both general neurodiversity dimension and condition‐specific variability; offers framework for understanding co‐occurrence and heterogeneity
Azim et al. (2025)	Australian Autism Biobank (*n* = 886) + Child Behavior Research Clinic (*n* = 460)	Cluster analysis + factor analysis	Communication domain Emotional regulation domain Social interaction domain Behavioral control domain Cognitive functioning domain Restricted repetitive behavior domain Sensory domain Motor domain Daily functioning domain	Integrated findings revealed consistent patterns across domains, supporting the development of a unified transdiagnostic assessment scale capturing overlapping characteristics across autism, ADHD, and other NDD
Cleary et al. (2026)	1716 participants (TD, ASD, ADHD)	Heterogeneous mixture modeling	Six distinct behavioral clusters with profiles ranging from low impairment to severe, cross‐domain difficulties	All six clusters were transdiagnostic, containing individuals from multiple diagnostic categories; most severely impaired cluster comprised exclusively ASD and ADHD participants
Holmes et al. (2021)	805 children (CALM cohort)	Factor analysis	Phonological processing difficulties Processing speed deficits Executive functioning difficulties	Three core cognitive dimensions captured shared variance across children with varied NDD presentations
Jacobs et al. (2021)	176 children with ASD, ADHD, and OCD	Integrating multi‐modal neuroimaging with behavioral data	Four robust, transdiagnostic biotypes	Biotypes characterized by distinct patterns of cortical thickness, subcortical volume, and symptomatology that were not discernible through categorical comparisons
Jones et al. (2021)	227 children (CALM cohort)	Resting‐state fMRI + behavioral profiling	Three distinct behavioral profiles characterized by difficulties with: Hot executive function (emotion/reward) Cool executive function (cognitive control) Learning	Multivariate patterns of functional connectivity, particularly within networks supporting cognitive control, emotion, and social cognition, significantly predicted group membership
Mareva et al. (2023)	805 children (CALM cohort)	Community detection	Subgroup 1: Structural language difficulties Subgroup 2: Cool executive function deficits Subgroup 3: Hot executive function & pragmatic communication difficulties	Subgroups showed distinct patterns of academic achievement, socioemotional functioning, and white matter organization; better explained commonalities than traditional diagnostic labels
Márquez‐Caraveo et al. (2021)	Clinically well‐characterized Mexican sample with intellectual disability, ASD, and ADHD (*n* = 74)	Latent class analysis of WISC‐IV profiles	Four‐cluster solution cutting across diagnoses	Children with same diagnostic label often fell into different cognitive subgroups; for example, those with ASD were distributed across three distinct classes based on working memory and perceptual reasoning scores

Of course, because the transdiagnostic approach is data‐driven, the stability of identified clusters across samples may raise concerns. However, this is not necessarily a limitation; rather, it reflects another fundamental difference from traditional diagnostic models. The aim of the transdiagnostic approach is not to establish a single, universally applicable clustering solution, but to delineate meaningful patterns of difficulty within specific samples based on the research questions and objectives at hand. In other words, the diversity of clusters across studies is a feature of this approach. That said, methodological strategies such as using large‐scale samples, integrating multi‐modal data (e.g., cognitive, behavioral, neural, and genetic), and applying advanced ML techniques can enhance the consistency and replicability of findings across studies (Astle et al. 2022). These issues will be explored in greater detail in subsequent sections.

Another frequently raised concern pertains to the potential for fundamental differences in etiology across NDD categories. In response, it is important to recognize, as several researchers have noted, that the relationship between phenotype and etiology is complex and multifaceted (e.g., Diao et al. 2026; Ding 2026). On the one hand, the transdiagnostic approach does not preclude the investigation of etiology; indeed, it may offer a more productive pathway. As the studies reviewed above suggest, there may be shared mechanisms across NDD that bring us closer to understanding the fundamental architecture underlying these conditions. On the other hand, by attending to the multiple possible relationships between etiology and phenotype, the transdiagnostic framework facilitates a more productive integration of theory and practice. The goal is not to assert a single cause for all difficulties, but to provide a multi‐level system map within which diverse causal pathways operate. By focusing on shared dimensions and their interactions, we can better understand how heterogeneous etiologies converge on common patterns of difficulty and, conversely, how a single etiological pathway can diverge into varied phenotypic outcomes.

To summarize, transdiagnostic studies of children with various difficulties or diagnoses share several features. First, children showing difficulty in certain areas (learning, memory, attention, etc.) with or without a diagnosis are included in the study. Second, data were collected from multiple sources. Finally, data analysis often incorporates data‐driven and theory‐driven methods. In addition to the transdiagnostic perspective, the dimensions or factors to be included should also be an important part of the approach. Previous studies have mainly focused on the behavioral and neural aspects of children with NDD symptoms, which are closely related to the diagnostic criteria. However, focusing solely on the neurocognitive domain may be limiting, as other factors, such as psychological and environmental factors, can also play an important role in the symptoms observed in children with NDD (Norwich 2016). Hence, a comprehensive framework is needed to fully examine how the different dimensions interact within the NDD population. The biopsychosocial model is a conceptual framework that considers both the medical model (which views disability as a result of impairment) and the social model (which holds that disability is a response to society's inflexibility toward individual differences) when examining disability (Anderberg 2005). This view of disability will be reviewed in the next section.

## 3 The Important Psychological and Socio‐Ecological Factors

There are two main views of disability that generally affect how people understand it: the medical model, which focuses on individual impairments, and the social model, which emphasizes the impact of societal barriers (Shakespeare 2008). The medical model of disability suggests that the challenges faced by individuals and the barriers they encounter are largely attributable to their impairments. This perspective focuses on the individual's deficits rather than considering the broader social and environmental factors that may contribute to their experiences (Shakespeare 2006). However, this type of view has been critiqued by people with disabilities from the disability movement in the UK, who advocated an opposite model, which is the social model view of disability. The social model view of disability emphasizes that disability is not solely a result of an individual's impairments but rather a consequence of the interaction between individuals and their environments, including societal attitudes and physical barriers (Mitra 2006; Oliver 2013).

However, this dichotomous way of viewing disability still could not describe the actual situation that people with NDD experience, given that NDD is influenced by factors from multiple domains (Homberg et al. 2016). Although ongoing research continues to advance understanding of the underlying etiological mechanisms, no single unifying cause has yet been elucidated (Hodges et al. 2020). There is a need to combine medical and social perspectives, considering an individual's functioning as a complex interaction among biological, psychological, and social aspects (Norwich 2016). Hence, the biopsychosocial model of disability, which underlies the International Classification of Functioning, Disability and Health (ICF), is more suitable for addressing the needs of children with NDDs, as it provides a comprehensive framework that integrates multi‐domain factors to inform tailored interventions and support strategies (Dalgleish et al. 2020).

The psychosocial factors encompass the psychological, social, and cultural context of an individual's daily life and may have a powerful impact on mental health and cognitive/physical functioning (Bell 2017; González‐Rodríguez et al. 2024). A great amount of research evidence supports the association between psychological problems and ADHD/ASD symptoms. Emotional dysregulation, for instance, has been increasingly recognized as a core feature of ADHD rather than merely a secondary consequence. Shaw et al. (2014) found that deficits in emotion regulation were present in a substantial proportion of children with ADHD and were associated with greater impairment in peer relationships, academic functioning, and family dynamics, independent of the core symptoms of inattention and hyperactivity. Similarly, children with ASD frequently experience elevated rates of anxiety and depression, which have been shown to compound social communication difficulties and exacerbate restrictive and repetitive behaviors (White et al. 2009; Vasa et al. 2020). A meta‐analysis by van Steensel et al. (2011) confirmed that anxiety disorders are among the most common comorbid conditions in youth with ASD, with prevalence rates significantly higher than in the general population.

Furthermore, for children with ADHD, low self‐esteem and negative self‐concept have been found to be associated with poorer academic achievement and increased risk of later mood disorders, suggesting that psychological characteristics mediate the long‐term trajectory of the disorder (Bussing et al. 2000; Harpin et al. 2016). In ASD, the presence of internalizing symptoms such as anxiety has been linked to greater social impairment and reduced quality of life, underscoring that psychological factors contribute to functional outcomes beyond the core diagnostic features (Chang et al. 2012). Furthermore, longitudinal studies have demonstrated bidirectional relationships between NDD symptoms and psychological factors. For example, children with ADHD who exhibit oppositional behaviors often experience escalating conflict with parents and teachers, which in turn exacerbates both behavioral difficulties and emotional distress (Johnston and Mash 2001). Similarly, in ASD, anxiety symptoms have been shown to intensify social withdrawal, reducing opportunities for social learning and thereby maintaining or worsening social deficits (Wood and Gadow 2010). A more recent longitudinal study by Duvekot et al. (2018) found that higher levels of anxiety symptoms predicted increased social communication impairment over time in children with ASD, further supporting the directional influence of psychological factors on core ASD symptoms.

In the context of learning difficulties, children with reading and spelling problems consistently exhibit higher rates of both externalizing (e.g., inattention, hyperactivity) and internalizing (e.g., anxiety, withdrawal) problems than their typically developing peers (Vieira et al. 2024). Liu et al. (2023) demonstrated that among first‐grade Chinese children, reading self‐efficacy contributed to word reading indirectly through its effect on reading‐specific cognitive abilities (e.g., vocabulary and morphological awareness). Arrow et al. (2025) further identified distinct learner profiles among beginning readers, with approximately 20% of their sample characterized by low reading self‐efficacy and concurrent behavioral difficulties. These profile differences were evident at school entry and continued to be associated with literacy outcomes across the first 3 years of schooling, demonstrating that psychological factors are measurable early and have sustained implications for literacy development. Chung et al. (2024) investigated Chinese adolescents with and without dyslexia and found that readers with dyslexia reported higher levels of both general anxiety and reading‐specific anxiety than typical readers, as well as lower levels of reading self‐concept. Importantly, after controlling for cognitive factors such as rapid naming and verbal working memory, reading self‐concept remained uniquely associated with word reading and reading fluency for both groups, while reading anxiety and reading self‐concept were uniquely associated with reading comprehension. These findings demonstrate that affective factors contribute to reading outcomes independently of well‐established cognitive predictors.

Collectively, these findings demonstrate that psychological factors such as emotional regulation, self‐concept, and anxiety are not peripheral but actively shape symptom expression, functional impairment, and developmental trajectories across diverse NDD symptoms. As such, understanding the full complexity of NDD outcomes requires attending to these psychological characteristics alongside core neurocognitive profiles.

The key socio‐ecological factors that could affect children could include family functioning and parenting, family socioeconomic status, peer relationships, school environment, trauma, and adverse childhood experiences, etc. In the context of inclusive education, previous research has shown a close relationship between NDD symptoms and socio‐ecological factors (e.g., Operto et al. 2021; Senechal 2006). However, what is often underscored in the literature, yet warrants repeated emphasis, is that these psychosocial and socio‐ecological factors are as critical as, if not more significant than, neurocognitive factors for understanding the difficulties of children with NDD and for providing more effective support to them and their families.

A growing body of evidence highlights how these “non‐cognitive” factors can profoundly shape child outcomes across various domains of development. For instance, parental mental health has been established as a significant risk factor for child psychopathology. A comprehensive meta‐analysis by Robinson et al. (2024), synthesizing 58 studies over nearly four decades, found that parental stress and parental depression were significantly associated with an increased risk for ADHD in children, both in terms of diagnosis and symptom severity (Robinson et al. 2024). A systematic review by Martin et al. (2019) examining the association between child sleep problems and family functioning found that for children with ASD, there were small to large associations between parenting stress and child sleep problems across multiple studies. Particularly, the authors highlight the bidirectional relationships between the symptoms on the child and parent sides, suggesting that the issues should be holistically considered in the ecosystem, rather than simply treating them as predictors or outcomes (Martin et al. 2019). As Crowell et al. (2019) articulate in their review on parenting behavior and child development in ASD, the impairments in social relatedness characteristic of these children can strain parent–child interactions, and parental stress can have negative transactional effects that impede children's development (Crowell et al. 2019).

Some researchers argue that introducing psychosocial factors into (trans)diagnostic models may obscure the core neurocognitive mechanisms of NDDs, as they are traditionally defined by cognitive‐neural deficits (Pennington 2006; Sonuga‐Barke and Halperin 2010). While this critique holds merit from a conventional diagnostic perspective, where psychosocial factors are often viewed as secondary consequences or influential factors, the biopsychosocial model challenges this framework. From this perspective, the transdiagnostic approach should not merely replicate the nature of the conventional diagnostic approach by merely considering the commonalities across NDDs, but rather reflect the purpose/function of diagnosis and move forward.

As emphasized by the World Health Organization (WHO)'s Children and Youth Version (ICF‐CY), the goal of classification is “not a diagnosis for a child, but a profile of its functioning” (WHO 2007, 19), which depends on the interplay of cognitive, psychological, and ecological factors. That is to say, a neurocognitive‐based (trans)diagnosis may tell us the neurobiological origins of the problems, but is not competent to depict the full profile of each individual child. Although they may not be the initial base of NDDs, the psychosocial factors could obviously worsen the situation. Based on the findings of their review study, Wu et al. (2023) confirmed the high prevalence of psychological problems in children with ASD or ADHD and revealed that these psychological problems would worsen the symptoms of these children. As indicated by Scattolin et al. (2022), poor family environments would limit young children from fulfilling their developmental potential, while, on the other hand, effective parent–child relationships would critically reduce NDD symptoms and their impact. Crowell et al. (2019) indicated in their research that high levels of parental psychosocial stress would create a suboptimal family environment, while the stressful cycle in the family would hinder the effectiveness of interventions and worsen the core symptoms in children with NDDs. Thus, from our point of view, psychosocial factors are equally critical to cognitive ones in understanding and supporting children with NDDs, and the transdiagnostic approach and biopsychosocial model should be considered at the same time for informing inclusive education research and practice.

## 4 Future Direction: The Transdiagnostic‐Biopsychosocial Approach

Building on the synthesis above, we propose a future research and practice agenda that integrates the transdiagnostic approach with the biopsychosocial model, that is, the Transdiagnostic‐Biopsychosocial Approach (TBPS), to address the limitations of current NDD classification and support. The current transdiagnostic approach emphasizes the identification of shared dimensions, mechanisms, and risk/protective factors across different types of NDD that are not fully reflected in the traditional diagnostic categories (Astle et al. 2022). Despite its significant movement to break the conventional categorically labelling system by emphasizing the commonalities across children with diverse NDD, they mainly focus on the neuro‐cognitive factors. At the same time, the biopsychosocial model recognizes that child functioning and development are shaped by the dynamic interplay of biological, psychological, and social/environmental influences, and thus emphasizes the importance of both cognitive and non‐cognitive factors in depicting children's development profiles, whether they are typical or with NDD. However, the commonalities across various NDD were not explicitly articulated in the biopsychosocial model.

**FIGURE 1:**
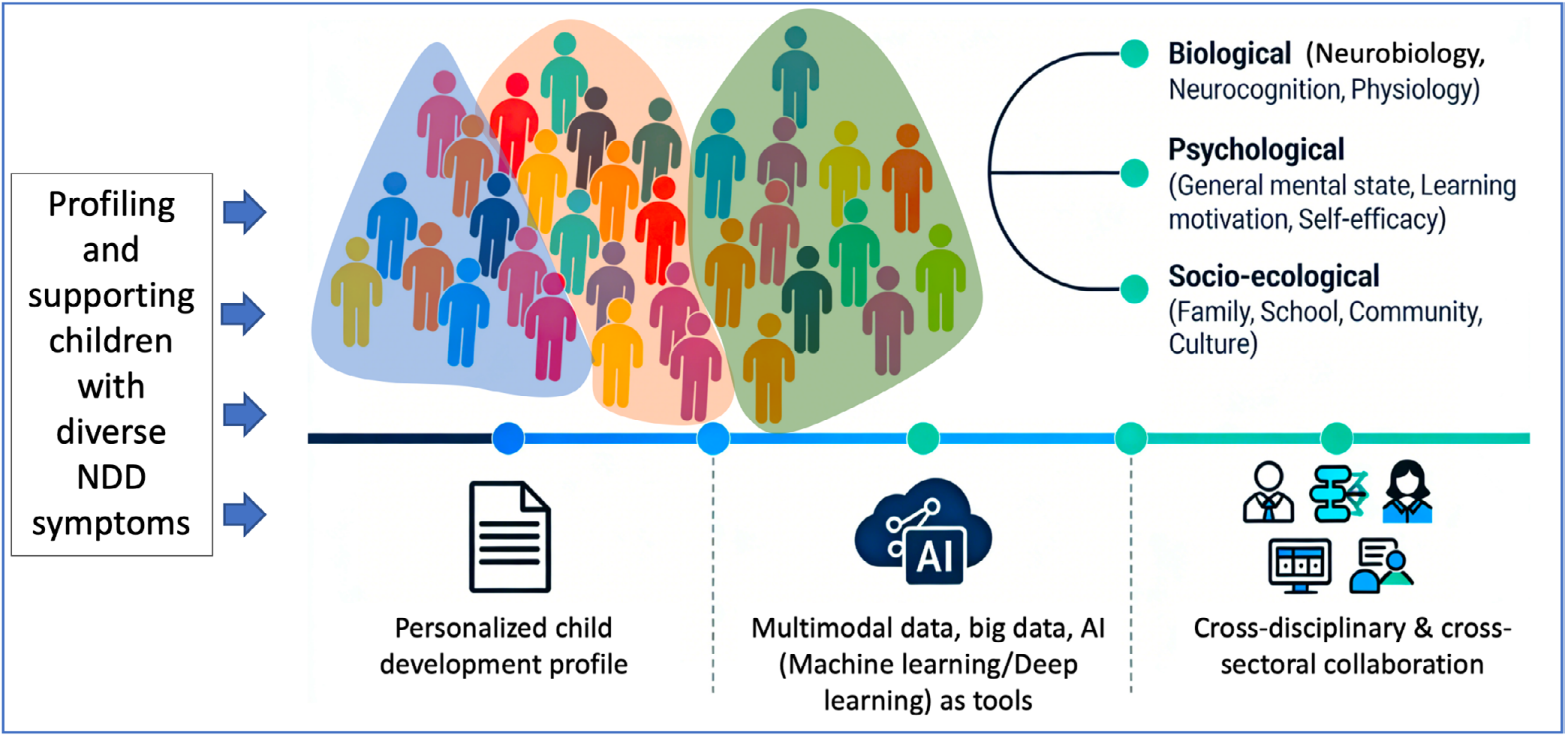
Schematic diagram of the Transdiagnostic Biopsychosocial (TBPS) Framework.

By combining these two perspectives, TBPS promotes a holistic, nuanced understanding of NDD, one that moves beyond narrow symptom checklists to consider the full spectrum of influences on a child's life and learning trajectory. This integrated framework is not intended to replace neurocognitive inquiry but to contextualize it, offering a more ecologically valid and functionally oriented pathway to understanding and supporting children with diverse neurodevelopmental profiles. Please refer to Figure 1 for a visualized illustration of the TBPS framework.

One core premise of TBPS is that a child's functioning is an emergent property of dynamic interactions between biological predispositions, psychological processes, and social environments. This TBPS approach is deeply aligned with the WHO's ICF, which shifts the focus from diagnosis to functioning. It also mirrors the “whole‐person approach” championed in inclusive education philosophy (Saito and Akiyama 2022), which calls for supporting the entire child in their ecological context, not just remediating their deficits. The TBPS provides the mechanistic model and empirical rigor to operationalize this philosophy, specifying which biological, psychological, and social factors interact and how those interactions impact educational participation and well‐being.

Building on the empirical evidence reviewed in the preceding sections, future research adopting the TBPS framework should systematically attend to a set of core factors across the three domains. These factors, summarized below, are not exhaustive but represent those most consistently identified as critical for understanding and supporting children with neurodevelopmental differences.

### 4.1 Neurocognitive Domain

Within the neurocognitive domain, both learning‐specific and general cognitive abilities warrant attention. Learning‐specific factors include literacy‐related skills, such as phonological processing, morphological awareness, and orthographic knowledge (Holmes et al. 2021; Liu et al. 2023), as well as arithmetic‐related skills, including number sense, magnitude comparison, and arithmetic fact retrieval (Butterworth et al. 2011; Träff et al. 2017). General cognitive abilities encompass executive functions (e.g., inhibition, working memory, cognitive flexibility), processing speed, and metacognition (Holmes et al. 2021; Mareva et al. 2023). These cognitive dimensions have been shown to capture shared variance across children with varied NDD presentations and to predict academic and functional outcomes (Holmes et al. 2021).

### 4.2 Psychological Domain

The psychological domain encompasses both general emotional and behavioral functioning and learning‐specific psychological factors. General psychological status includes internalizing problems (e.g., anxiety, depression, withdrawal) and externalizing problems (e.g., aggression, conduct difficulties), which are prevalent among children with NDDs and have been shown to interact with neurocognitive vulnerabilities (Azim et al. 2025; Cleary et al. 2026). Learning‐specific psychological factors, such as academic motivation, reading self‐concept, and academic self‐efficacy, are equally important. As demonstrated in studies of literacy development, these motivational factors contribute uniquely to learning outcomes, even after controlling for well‐established cognitive predictors (Chung et al. 2024; Liu et al. 2023).

### 4.3 Social‐Ecological Domain

The social‐ecological domain requires attention to multiple layers of environmental influence, consistent with bioecological models of development (Bronfenbrenner and Morris 1998). At the family level, key factors include parenting style, parent–child relationship quality, home literacy environment, and parental mental health and stress (Crowell et al. 2019; Liu et al. 2023; Robinson et al. 2024). At the school level, teacher‐student relationships, peer relationships, school climate, and the availability of inclusive support services are critical (Azim et al. 2025; Tyldesley‐Marshall et al. 2025). In multicultural circumstances, cultural factors, including cultural attitudes toward disability, language of instruction, and family expectations, should also be considered, as these shape both the expression of NDD symptoms and the availability and acceptability of support (Huang et al. 2013; Sun et al. 2019).

### 4.4 Interplay Across Domains

Crucially, these domains are not independent but dynamically interact over time. For instance, a child's reading self‐efficacy (psychological) may be shaped by the home literacy environment (social‐ecological) and, in turn, influence their engagement with reading tasks, which shapes their reading‐related brain development (neurocognitive) over time. Conversely, neurocognitive vulnerabilities may increase a child's susceptibility to negative environmental influences, such as parenting stress or peer rejection, creating cascading effects across development (Crowell et al. 2019; Martin et al. 2019). Future research adopting the TBPS framework should therefore prioritize longitudinal designs and analytical approaches capable of capturing these bidirectional, dynamic relationships, moving beyond cross‐sectional, variable‐centered analyses to person‐centered methods that can reveal how factors across domains coalesce into meaningful profiles for individual children.

In some existing studies following the transdiagnostic approach in the field of NDD, albeit their focus on the commonalities in the domain of neurocognition, the functions of psychological and ecological factors were considered (e.g., Apperly et al. 2024; Cleary et al. 2026; Mareva et al. 2023). These studies demonstrate that it is both feasible and empirically illuminating to position psychological and ecological factors as central to transdiagnostic subgroup identification, offering a methodological template for operationalizing the TBPS framework's call to capture the dynamic interplay of biological, psychological, and social systems in shaping developmental outcomes.

A successful TBPS agenda demands collaboration across disciplines (Sun et al. 2026). This could include genetics, neuroscience, psychiatry, psychology, education, social work, rehabilitation sciences, and more. Each discipline brings unique expertise and methods for understanding different facets of NDD, from molecular pathways and neural circuits to classroom dynamics and family systems. As Dalgleish et al. (2020) note, the diagnostic paradigm's limitations have inspired the development of large‐scale consortia such as the Research Domain Criteria (RDoC) and the Hierarchical Taxonomy of Psychopathology (HiTOP), which represent the collaborative efforts of many international scientists from multiple disciplines. Joining and supporting these consortia is essential for advancing transdiagnostic science (Dalgleish et al. 2020).

Similarly, cooperation across sectors, for example, healthcare, education, social services, and community organizations, is essential to bridge the gap between assessment, intervention, and long‐term support. This cross‐sectoral collaboration has been emphasized in many societies during the implementation of inclusive education (e.g., UNESCO 2020). The TBPS philosophy can be promoted through these established cross‐disciplinary and cross‐sectoral collaboration platforms, with the purpose of optimizing the profiles of diverse children and bettering their lives rather than identifying and eliminating the problems. In essence, TBPS provides a unifying framework that aligns scientific inquiry with person‐centered, ecologically valid practice, ensuring that children with NDD are understood and supported in all their complexity.

Besides in empirical studies, this TBPS framework has already begun to influence practice through integrated, cross‐sectoral service models, although not as a clear and systematic structure. That is to say, the current emphasis on collaboration and cooperation among the medical, educational, community, and family systems in supporting children with special educational needs (SEN) reflects the practical application of TBPS principles (Castro‐Kemp and Samuels 2022; Tyldesley‐Marshall et al. 2025). Such cross‐sectoral collaboration recognizes that a child's developmental outcomes cannot be adequately addressed by any single system in isolation.

As Tyldesley‐Marshall et al. (2025) identified in their systematic review of partnership working in the Special Educational Needs and Disabilities (SEND) services, five key ingredients for effective collaboration include participation and legitimacy, personalization and consultation with families, respectful communication, preparation for partnership roles, and working across professional and organizational boundaries. Healthcare professionals bring expertise in biological and neurocognitive assessment; educators contribute understanding of learning processes and classroom dynamics; community organizations provide social support and recreational opportunities; and families offer indispensable knowledge of the child's daily functioning, history, and values.

Protocols such as the “F‐words for Child Development,” focusing on Functioning, Family, Fitness, Fun, Friends, and Future, are good examples of the practical implementation of the TBPS framework that have been widely adopted internationally to operationalize a strengths‐based, holistic approach to child disability, shifting the focus from deficits to abilities and participation (Rosenbaum and Gorter 2012). These successful initiatives share a common foundation: they recognize that a child's functioning is shaped by the dynamic interplay of biological, psychological, and social factors, and they deliberately structure collaboration across the medical, educational, community, and family systems to address the whole child in context, without specifically focusing on diagnostic labels. The task ahead is to build upon these prototypes, refining and extending them through further research and development to realize the full potential of the TBPS framework for understanding and supporting children with NDD.

Last but not least, methodological consideration is critical for practically implementing the TBPS framework. Conventionally, comprehensive profiling of a child's needs across multiple domains requires extensive, time‐consuming assessment batteries, posing practical barriers to wide‐scale adoption. Recent advances in artificial intelligence (AI) and digital health technologies offer a promising solution. AI‐based assessment platforms can efficiently integrate data from a variety of sources, such as eye‐tracking, voice and gesture analysis, video observation, digital games, and parent/teacher reports, to generate multidimensional profiles with minimal burden on the child and family. Advanced analytic methods, such as ML and deep learning (DL) networks, have been applied in transdiagnostic studies to expand the literature and offer new perspectives. Unlike conventional methods, ML/DL does not rely on specific assumptions, making it suitable for handling large, multidimensional datasets, imbalanced data, missing values, and nonlinear relationships. Recent ML‐based studies have sought to validate the reliability and accuracy of ML in identifying and classifying critical factors or at‐risk populations (e.g., Al‐Saoud et al. 2025; Mareva et al. 2023). In their review study, Mohamed et al. (2026) examined the application of ML and DL across NDD, highlighting successful applications using diverse data modalities including neuroimaging, EEG, clinical and sociodemographic data, genetic profiles, and wearable sensor data. They reported high diagnostic accuracy across conditions, with models for ASD achieving 89%–99% accuracy, ADHD models reaching up to 99%–100% accuracy, and AI‐based systems for learning disabilities and motor disorders demonstrating performance comparable to or exceeding traditional clinician‐led methods. The review concludes that AI tools demonstrate strong potential as clinical adjuncts for early detection and personalized support.

Despite these promising advances, significant challenges remain. As Mohamed et al. (2026) highlight, current applications of AI in this field are constrained by limited transparency in modelling processes, concerns about data security and algorithmic bias, and a lack of large, diverse datasets to ensure the generalizability of findings. These limitations notwithstanding, the integration of AI tools represents an indispensable direction for the future of NDD research and practice. In particular, if the TBPS framework is to be effectively implemented across both scientific inquiry and real‐world applications, AI technologies will serve as a crucial methodological foundation, enabling the scalable, multidimensional profiling that the framework demands.

With cross‐disciplinary and cross‐sector collaborations and the application of AI techniques, several key questions should be addressed by future research in this direction. For example, which psychosocial factors (e.g., parenting, peer relationships, school climate, socioeconomic status) most strongly interact with neurocognitive vulnerabilities to shape outcomes for children with NDDs across different developmental periods? How do these factors moderate or mediate the impact of shared transdiagnostic mechanisms? What are the most efficient and sensitive ways to measure these influences in real‐world settings? How can interventions be tailored to address each child's unique biopsychosocial profile rather than applying one‐size‐fits‐all protocols? Addressing these questions will require not only continued methodological innovation but also sustained commitment to the cross‐disciplinary and cross‐sector collaborations that are essential for realizing the full potential of the TBPS framework.

## 5 Conclusion

Conclusively, the integration of transdiagnostic and biopsychosocial approaches represents a paradigm shift that is required in the field of NDD and for the further success of inclusive education. That is to say, the TBPS approach offers a unifying framework that aligns cutting‐edge science with person‐centered, ecologically grounded practice, moving the field beyond the limitations of diagnostic labels towards a future where every child is understood as a whole person shaped by the unique interplay of neurobiological, psychological, and social‐ecological factors.

The implementation of this approach demands collaborative effort in at least four key directions. First, we should foster cross‐disciplinary and cross‐sectoral partnerships, bringing together researchers, clinicians, educators, families, and community organizations as equal partners in both inquiry and support. Such collaborations are essential for bridging the gap between research and practice, ensuring that insights from neuroscience, psychology, and education translate into meaningful improvements in children's daily lives. Second, sustained investment in AI‐enabled, multidimensional assessment tools is critical to efficiently and ethically capture each child's dynamic profile. We should develop mechanisms for interpreting and sharing data across sectors in a fair and secure manner, enabling these tools to function to their full potential.

Third, longitudinal studies are needed to disentangle the complex relationships among factors across different domains and to develop individualized models for children with specific profiles. Making the situation more complicated is not the purpose of the TBPS approach. The complexity entailed by integrating a wide array of factors from multiple domains, together with the progressive dissolution of traditional diagnostic boundaries, should be understood as an unavoidable initial stage of this research endeavor. These longitudinal and individualized models could help update the relevant theories and find a way out for this TBPS approach. Such work will require not only methodological sophistication but also deep engagement with communities to ensure that research questions reflect the priorities of families and practitioners. Fourth and finally, systemic policy and practice reform is expected. We must advocate for a fundamental shift from diagnosis‐based resource allocation to a needs‐based, individualized support system that responds to each child's unique profile of strengths and challenges. Along with the development of our societies, the “melt” of inclusive education into the broader “education” should be an ideal future solution, ensuring that all children's diversities are fully recognized and supported.

The path forward is neither simple nor short, but the growing consensus across disciplines and the emergence of innovative, collaborative models give reason for optimism. By committing to this integrated vision, we can move beyond the constraints of categorical thinking and build a world that truly sees, supports, and celebrates every child in all their natural complexity.

## Funding

This work was supported by the Multi‐Disciplinary Research Capacity Building Scheme Grant of the Education University of Hong Kong, 1‐32‐04A29.

## Conflicts of Interest

The authors declare no conflicts of interest.

## Data Availability

The authors have nothing to report.
